# Microbial diversity in intensively farmed lake sediment contaminated by heavy metals and identification of microbial taxa bioindicators of environmental quality

**DOI:** 10.1038/s41598-021-03949-7

**Published:** 2022-01-07

**Authors:** María Custodio, Ciro Espinoza, Richard Peñaloza, Tessy Peralta-Ortiz, Héctor Sánchez-Suárez, Alberto Ordinola-Zapata, Enedia Vieyra-Peña

**Affiliations:** 1 Centro de Investigación de Medicina en Altura y Medio Ambiente, Facultad de Medicina Humana, Universidad Nacional del Centro del Perú, Av. Mariscal Castilla N° 3909, Huancayo, Peru; 2 Facultad de Ingeniería Pesquera y Ciencias del Mar, Universidad Nacional de Tumbes, Calle Los Ceibos S/N, Puerto Pizarro, Tumbes Peru; 3 Departamento Académico de Medicina Veterinaria y Zootecnia, Facultad de Ciencias Agrarias, Universidad Nacional de Tumbes, La Cruz S/N, Tumbes, Peru

**Keywords:** Ecology, Microbiology, Environmental sciences

## Abstract

The cumulative effects of anthropogenic stress on freshwater ecosystems are becoming increasingly evident and worrisome. In lake sediments contaminated by heavy metals, the composition and structure of microbial communities can change and affect nutrient transformation and biogeochemical cycling of sediments. In this study, bacterial and archaeal communities of lake sediments under fish pressure contaminated with heavy metals were investigated by the Illumina MiSeq platform. Despite the similar content of most of the heavy metals in the lagoon sediments, we found that their microbial communities were different in diversity and composition. This difference would be determined by the resilience or tolerance of the microbial communities to the heavy metal enrichment gradient. Thirty-two different phyla and 66 different microbial classes were identified in sediment from the three lagoons studied. The highest percentages of contribution in the differentiation of microbial communities were presented by the classes Alphaproteobacteria (19.08%), Cyanophyceae (14.96%), Betaproteobacteria (9.01%) y Actinobacteria (7.55%). The bacteria that predominated in sediments with high levels of Cd and As were Deltaproteobacteria, Actinobacteria, Coriobacteriia, Nitrososphaeria and Acidobacteria (Pomacocha), Alphaproteobacteria, Chitinophagia, Nitrospira and Clostridia (Tipicocha) and Betaproteobacteria (Tranca Grande). Finally, the results allow us to expand the current knowledge of microbial diversity in lake sediments contaminated with heavy metals and to identify bioindicators taxa of environmental quality that can be used in the monitoring and control of heavy metal contamination.

## Introduction

Lake environments are fragile ecosystems that have been experiencing strong anthropogenic pressure and limited natural pressure. Anthropogenic stressors such as intensification of water use, overexploitation of hydrobiological resources, nutrient inputs, hydrological stress, dumping of agricultural, urban and industrial waste, in addition to natural stressors, are generating negative effects on these ecosystems^1,2^. Contamination of aquatic ecosystems by heavy metals, such as Cd, Pb, Hg and Ni, is a widespread concern due to their toxicity, persistence, non-degradability and tendency to accumulate in their various compartments^3–5^. In sediments, microbiota play a fundamental role in the biogeochemical transformations of nutrients and contaminants, and mediate the uptake, accumulation, release and transfer of heavy metals^6–9^.

Heavy metals in sediments under favorable conditions can be released to the water column^10^ and influence microbial abundance and diversity^11^. Microbial communities are strongly related to their surrounding environments and are sensitive to environmental changes caused by anthropogenic factors^12–14^. Microbial communities may undergo significant changes in structure and function due to heavy metal contamination^15^ and influence the ecological functions of aquatic ecosystems. Bacteria and archaea, as the most abundant sedimentary organisms, play key roles in nutrient recycling, the breakdown of chemical compounds and water quality^16,17^. They can also produce anionic polymers capable of complexing metals. Understanding the cumulative effects of heavy metal pollution on the composition and structure of microbial communities and function helps to find ways to reduce and remediate the damage of heavy metal pollution in aquatic systems^18^.

Culture-independent molecular methods currently used to study environmental microbiota include gene analysis, functional gene inventory and direct sequencing of DNA recovered from environmental samples^19,20^. Metagenomic analysis methods have proven to be useful in sediment microbial ecology studies^21,22^. In addition, they are of great importance for elucidating the composition of microbial communities because they provide simultaneous insight into the phylogenetic composition and metabolic capabilities of uncultivated populations^23^. Gene fragments from individual sequencing reads and small assembled segments can be annotated and assigned to approximate phylogenetic garbage cans based on known reference genomes^24^.

Several studies document that freshwater sediments have received less attention compared to sediments from marine environments^25^. Others report that the composition of sediment microbial communities can be influenced by physicochemical factors such as temperature, stream flow, pH and nutrients^26^. It is recognized that microbial communities co-vary with the high concentrations of nutrients and metals in sediments^27^. However, information on microbial diversity in sediments from ponds used in intensive fish farming with different trophic status and metal content is scarce. Therefore, the assessment of microbial community composition can lead to a better understanding of intensively farmed aquatic ecosystems and be a useful tool for environmental monitoring and control. In this regard, and considering that lake environments under fish farming pressure need to be monitored not only at the trophic state and metal content level but also at the microbial diversity level due to the fundamental role of microorganisms in nutrient cycling, structure and function of the ecosystem. The objective of this study was to investigate microbial diversity in lake sediment contaminated by heavy metals using metagenomics and to identify microbial taxa that are bioindicators of environmental quality.

## Results

### Status of lake sediment contamination by heavy metals

**Figure 1:**
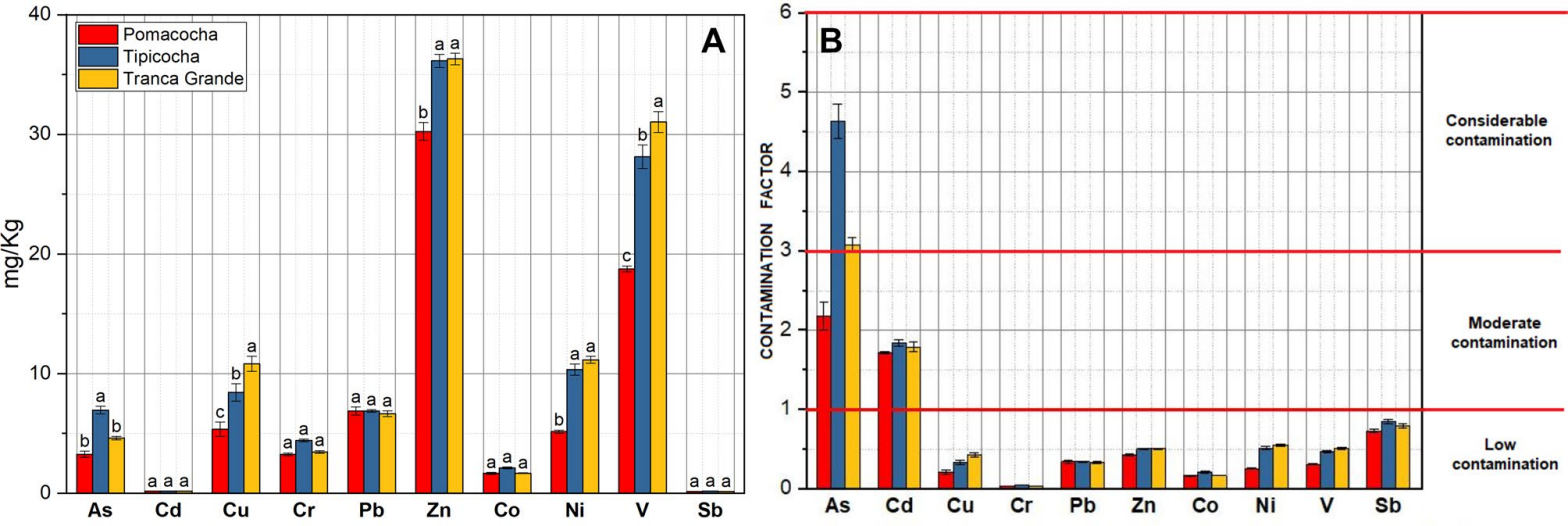
Comparison of the concentration of heavy metals in lake sediment according to the Kruskal–Wallis test (**A**). Heavy metal contamination factor (**B**).

Table 1 shows the descriptive statistics for heavy metals in lake sediment and mean values for the upper continental crust (UCC)^28^. The decreasing order of the mean concentrations of heavy metals in sediment was: Zn > V > Ni > Cu > Pb > As > Cr > Co > Cd > Sb. The highest mean concentration of Zn (36.30 ± 0.84 mg kg^‒1^), V (31.03 ± 1.53 mg kg^‒1^), Ni (11.16 ± 0.50 mg kg^‒1^), Cu (10.83 ± 1.10 mg kg^‒1^), Cr (3.45 ± 0.21 mg kg^‒1^) and Cd (0.18 ± 0.0. 011 mg kg^‒1^) was recorded in Tranca Grande lagoon and As (6.95 ± 0.57 mg kg^‒1^), Pb (6.88 ± 0.19 mg kg^‒1^), Co (2.12 ± 0.13 mg kg^‒1^) and Sb (0.17 ± 0.01 mg kg^‒1^) in Tipicocha lagoon. The lowest concentrations of heavy metals were recorded in the Pomacocha lagoon. The Kruskal–Wallis (KW) test revealed that the concentrations of As, Cu, Ni and V in sediment from the three lagoons differed significantly (p < 0.05) compared to the concentrations of Cd, Cr, Pb, Co and Sb which showed no significant differences (p > 0.05) (Fig. 1A). The present results were similar to the geochemical composition of bottom sediments from other lakes under similar fish pressure^29^ and to the UCC values, except for the concentration of As and Cd which were twice these values (1.5 and 0.098 mg kg^‒1^, respectively). The increase in As and Cd concentrations could be related to the fish farming activity carried out in floating cages.

**Table 1:** Descriptive statistics of heavy metals in lake sediment and mean values of heavy metals in the upper continental crust (mg kg^‒1^).

Heavy metal	Descriptive statistics	Pomacocha			Tipicocha			Tranca Grande			UCC
As	Range	2.94	−	3.78	6.44	−	7.56	4.48	−	4.90	1.5
	Mean ± SD	3.27	±	0.45	6.95	±	0.57	4.62	±	0.24	
Cd	Range	0.17	−	0.172	0.17	−	0.19	0.17	−	0.19	0.098
	Mean ± SD	0.17	±	0.002	0.18	±	0.01	0.18	±	0.01	
Cu	Range	4.39	−	6.45	7.30	−	9.81	9.69	−	11.87	25
	Mean ± SD	5.37	±	1.04	8.43	±	1.27	10.83	±	1.10	
Cr	Range	3.02	−	3.44	4.20	−	4.60	3.26	−	3.68	85
	Mean ± SD	3.25	±	0.21	4.41	±	0.20	3.45	±	0.21	
Pb	Range	6.21	−	7.40	6.67	−	7.03	6.30	−	7.13	20
	Mean ± SD	6.87	±	0.61	6.88	±	0.19	6.66	±	0.42	
Zn	Range	28.82	−	31.34	35.14	−	37.02	35.54	−	37.21	71
	Mean ± SD	30.24	±	1.29	36.14	±	0.95	36.30	±	0.84	
Co	Range	1.54	−	1.81	1.97	−	2.21	1.65	−	1.71	10
	Mean ± SD	1.67	±	0.14	2.12	±	0.13	1.68	±	0.03	
Ni	Range	4.97	−	5.42	9.46	−	11.08	10.62	−	11.61	20
	Mean ± SD	5.16	±	0.23	10.34	±	0.82	11.16	±	0.50	
V	Range	18.24	−	19.03	26.97	−	30.05	29.40	−	32.43	60
	Mean ± SD	18.75	±	0.44	28.13	±	1.68	31.03	±	1.53	
Sb	Range	0.14	−	0.15	0.16	−	0.18	0.15	−	0.17	0.2
	Mean ± SD	0.15	±	0.01	0.17	±	0.01	0.16	±	0.01

Figure 1B shows the contamination factor (CF) values for heavy metals in lake sediment. In Pomacocha, Tipicocha and Tranca Grande lagoons, the CF values of 80% of the heavy metals qualified as low contamination factor (CF < 1) and 20% as moderate (Cd, CF = 1–3) and considerable contamination factor (As, CF = 3–6). The increasing order of the range of moderate contamination of the lagoons (CF) by As was: Pomacocha (1.96–2.52) < Tranca Grande (2.99–3.27) < Tipicocha (4.29–5.04) and by Cd was: Pomacocha (1.70–1.75) < Tranca Grande (1.74–1.92) < Tipicocha (1.77–1.91) (Table S1).

### Microbial composition, diversity, and similarity profiles

A total of 32 phyla and 66 different classes of microorganisms were determined in sediment from three intensively farmed ponds. The most abundant phyla (> 1% of all sequences in all samples) were Proteobacteria (46.5–54.44%), followed by Cyanobacteria (8.40–18.64%), Actinobacteria (2.79–13. 30%), Bacteroidetes (8.68–10.31%), Firmicutes (3.96–6.24%), Acidobacteria (0.84–2.69%), Euryarchaeota (2.14–2.33), Chloroflexi (1.85–2.43%), and Ignavibacteriae (1.16–1.54%) (Table S2).

The similarity percentage procedure (SIMPER) analysis at the class level showed that the highest percentages of contribution in the differentiation of microbial communities were presented by the classes Alphaproteobacteria (19.08%), Cyanophyceae (14.96%), Betaproteobacteria (9.01%) and Actinobacteria (7.55%). Other classes with important contribution (> 1%) to the sedimentary microbial communities in high Andean lagoons were: Deltaproteobacteria (6.04%), Coriobacteriia (5.02%), Flavobacteriia (4.66%), Chitinophagia (4.04%), Clostridia (4.05%), Nitrospira (3.71%), Acidobacteria (2.84%), Methanomicrobia (2.28%), Gemmatimonadetes (1.88%), Gammaproteobacteria (1.78%), Dehalococcoidia (1.25%), Erysipelotrichia (1.13%) and Bacteroidia (1.02%) (Table S3). Members of the Archaea domain were also found in the lake sediments, with Euryarchaeota being the predominant phylum with an relative abundance ranging from 2.14 to 2.33%.

The number of microbial classes was similar in the three lagoons and was around 65 classes per lagoon (Table 2). The highest number of individuals was recorded in Tipicocha lagoon (112,995 ± 5016) followed by Tranca Grande (107,958 ± 2571) and Pomacocha (105,941 ± 1210). The Margalef and Chao-1 indices represent microbial richness and the Shannon and Simpson indices reflect microbial diversity. Margalef index revealed that Tipicocha is the lagoon with the lowest microbial richness. Chao-1 index has similar behavior as the Margalef index, with the value of the expected classes being higher in the Pomacocha lagoon, with 62.83 ± 2.02. While in the other two lagoons the microbial richness showed values around 60, indicating that Pomacocha is slightly more diverse, which could be due to the process of resilience due to the cessation of fish farming. The value of Simpson's index was similar in the three lagoons, varying from 0.89 ± 0.004 in Tranca Grande to 0.91 ± 0.002 in Tipicocha. Shannon index values in decreasing order were: 2.85 ± 0.02 (Tipicocha) > 2.64 ± 0.01 (Pomacocha) > 2.61 ± 0.04 (Tranca Grande).

**Table 2:** Diversity index mean of bacterial communities according to class by lagoon.

Lagoon	Individuals	Dominance_D	Margalef	Chao-1	Simpson_1-D	Shannon_H
	Mean (SD)	Mean (SD)	Mean (SD)	Mean (SD)	Mean (SD)	Mean (SD)
Pomacocha	105,941 (1210)	0.11 (0.002)	5.21 (0.05)	62.83 (2.02)	0.89 (0.002)	2.64 (0.01)
Tipicocha	112,995 (5016)	0.09 (0.002)	5.16 (0.10)	61.00 (1.00)	0.91 (0.002)	2.85 (0.02)
Tranca Grande	107,958 (2571)	0.11 (0.004)	5.12 (0.06)	61.17 (0.29)	0.89 (0.004)	2.61 (0.04)

**Figure 2:**
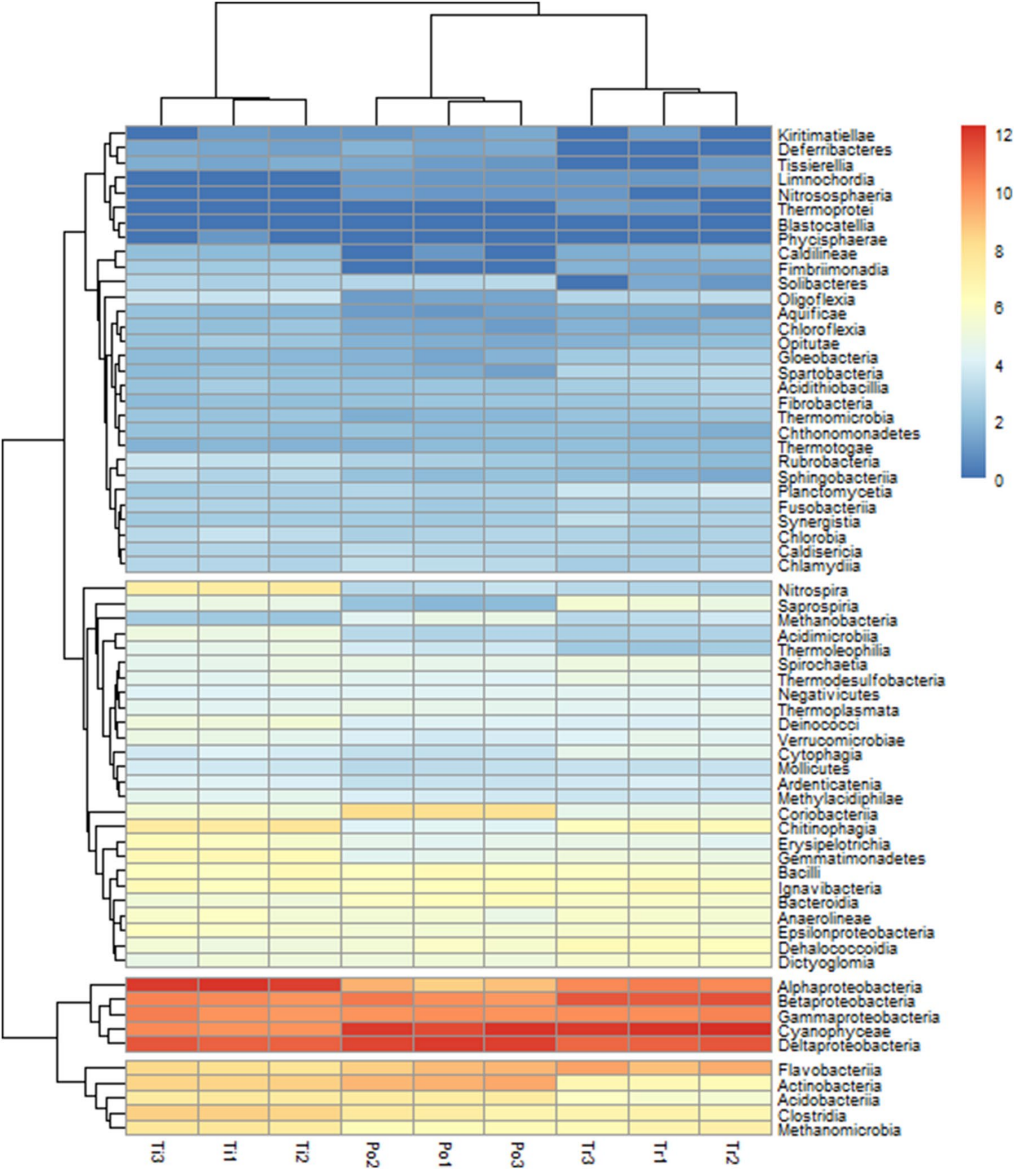
Heatmap showing the cluster (Bray Curtis distance) between sectors and based operational taxonomic units (OTU), transformed in fourth root. The color code indicates abundance, ranging from blue (low abundance) to red (high abundance).

The dendrogram based on the similarity of Euclidean distances at the class level showed that the sediment microbial communities of Pomacocha (Po), Tipicocha (Ti) and Tranca Grande (Tr) lagoons were divided into four groups (Fig. 2). The results obtained from the initial distance matrix showed that the distances of the first and second groups were significantly greater than the distances of the third and fourth groups with Euclidean distances (ED) close to 18. The first and second groups differed with values of 9, similarly the third and fourth groups (Fig. S1).

Based on abundance, the first group included the classes Methanomicrobia, Clostridia, Acidobacteria, Actinobacteria and Flavobacteria. The second and most frequent group consisted of Gammaproteobacteria, Betaproteobacteria, Deltaproteobacteria, Cyanophyaceae and Alphaproteobacteria. These microbial classes were the most representative and the ones that generated the greatest dissimilarity between the frequencies of classes found in the lagoons. Anaerolineae, Bacteroidia, Ignavibacteria, Bacilli, Gemmatimonadetes, Erysipelotrichia, Chitinophagia, Coriobacteriia, Methylacidiphilae, Ardenticatenia, Mollicutes, Cytophagia, Verrumicrobiate, Deinococci, Thermoplasmata, Negativicutes, Thermodesulfobacteria, Spirochaetia, Thermoleophilia, Acidimicrobia, Methanobacteria, Saprospriria and Nitrospira. The fourth group consisted of classes that are not representative and present uniform distribution in the sediment microbial communities of the three ponds. The result of the clustering also indicated that the first two microbial groups reveal that the microbial communities from the most dominant to the rarest were selected for their habitats.

### Microbial profiles of lake sediments with statistical differences

**Figure 3:**
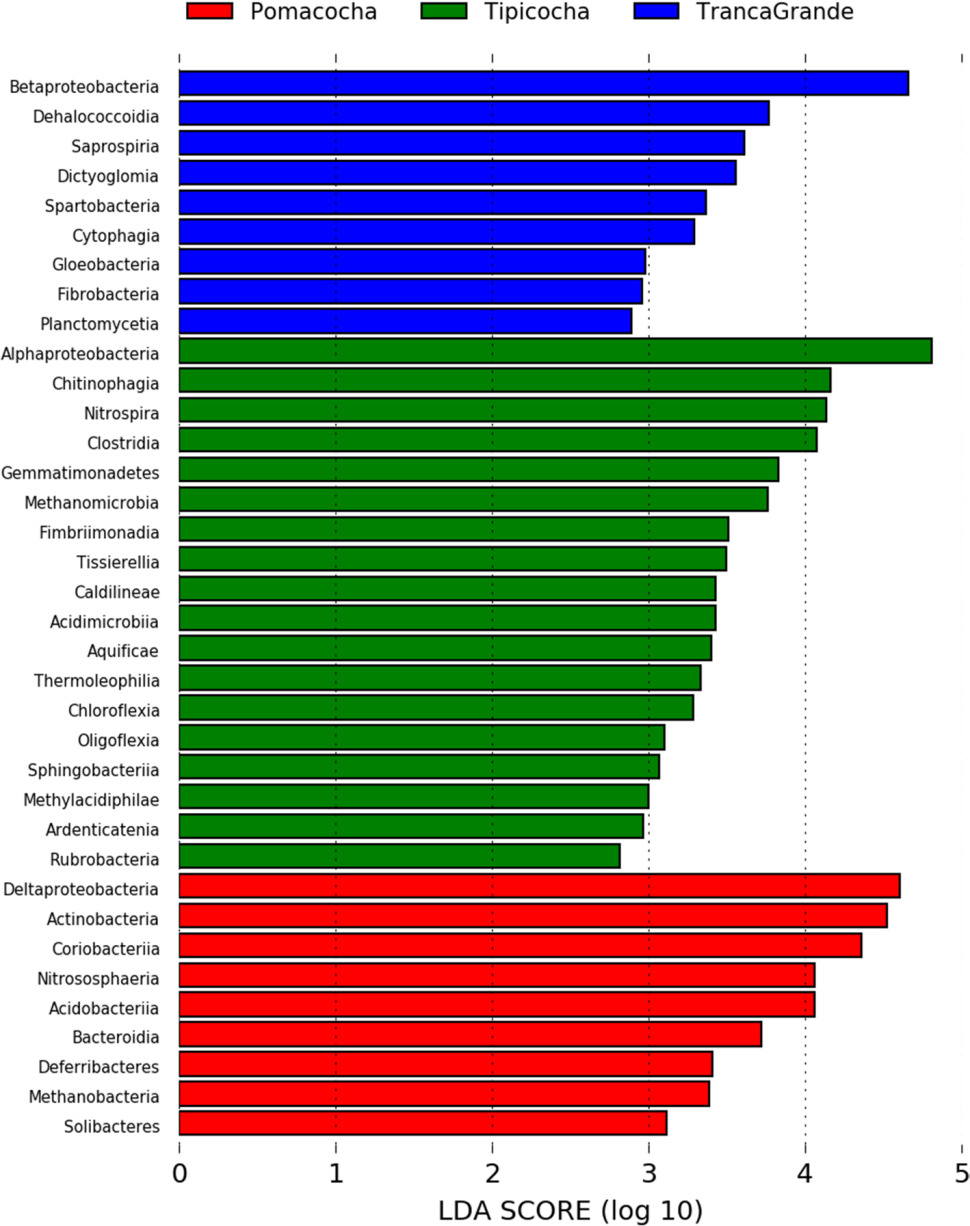
Linear discriminant analysis (LDA) effect size analysis (LefSe) at the operative taxonomic unit (OTU) level to compare microbiome profiles in sediments between lagoons.

The LefSe analysis was performed at the class level to examine the differences in the distribution of microbial classes according to the three lagoons evaluated. The discriminating and sediment-enriched microbial classes that presented a LDA value higher than 4.0 in Pomacocha lagoon corresponded to Deltaproteobacteria, Actinobacteria, Coriobacteriia, Nitrososphaeria and sediment Acidobacteria. In Tipicocha lagoon, Alphaproteobacteria, Chitinophagia, Nitrospira and Clostridia were predominant. In the Tranca Grande lagoon, the class that significantly enriched the sediment was Betaproteobacteria (Fig. 3).

The heavy metal contamination factor revealed that bacteria are prevalent in sediments with high levels of Cd and As. In this study, we suggest that Cd in sediments was probably derived from natural (lithogenesis, geological processes and natural weathering of rocks and minerals) and anthropogenic (fish farming, runoff from agricultural and mining areas, mainly) sources. Therefore, in lagoons with high Cd concentrations and fish farming pressure, these microbial classes were the most common, revealing their capacity to tolerate and adapt to contamination by this heavy metal. While the persistence of As denotes that it is an element that increases due to intensive fish farming activity. The microbial classes discriminated for Tipicocha lagoon would be the predominant ones in areas with high As levels. However, as the concentration of this element in particular tends to decrease, it favors the reaction and adaptability of the microbial classes discriminated in the Pomacocha lagoon.

### Effects of heavy metals on microbial communities

**Figure 4:**
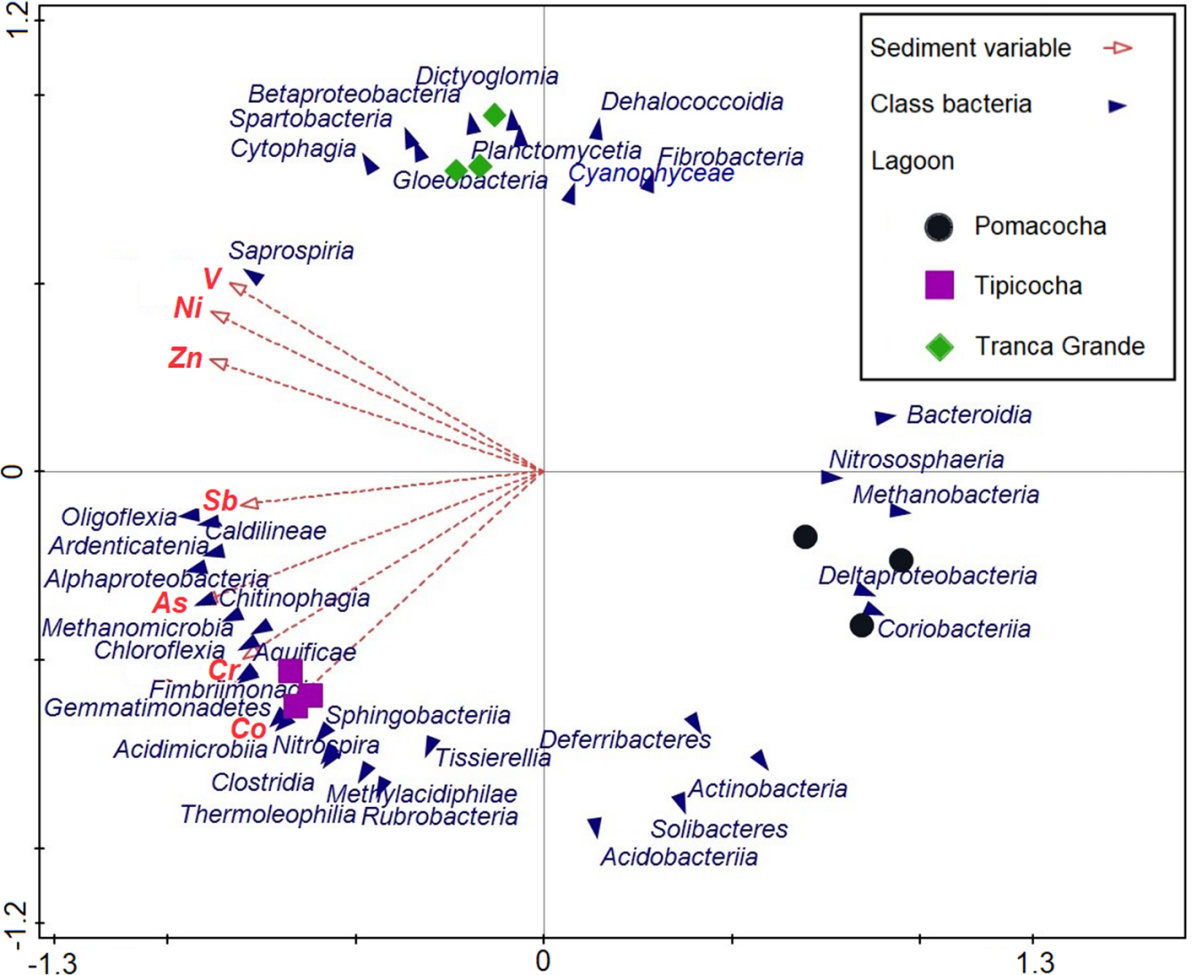
Redundancy analysis of the correlations between sediment heavy metal content (red lines) and microbial class distribution (blue arrow) across the study lagoons.

The forward stepwise forced RDA analysis with a total variance of 0.23 (Table S4), under an adjustment between biological and abiotic matrices, determined that As has a contribution to the explained distribution of both matrices of 50.1% of the total adjusted 91.61%. The difference in microbial classes between Tipicocha and Tranca Grande ponds was the response to the effect of V with a 39% contribution to the total explained distribution significantly (p < 0.05). These results reveal that these two elements were the most important factors that shaped the microbial community. The classes Alphaproteobacteria, Chitinophagia and Methanomicrobia were positively correlated with As, being in the Tipicocha lagoon a transcendental element explained by anthropogenic activities as a source of As in the lagoon. As, Sb, Cr and Co also showed positive associations to a lesser degree with the classes Oligoflexia, Ardenticatenia, Caldilineae, Methanomicrobia, Chloroflexia, Aquificae, Fimbriimonadia, Gemmatimonadetes, Sphingobacteriia, Acidimicrobiia and Nitrospira. On the other hand, V was positively and significantly correlated with the Saprospiria class. While the classes Bacteroidia Nitrososphaeria, Methanobacteria, Deltaproteobacteria and Coriobacteriia tended to be better expressed in environments with low concentrations of all the elements studied (Fig. 4).

## Discussion

Microbial communities play a fundamental role in the function of aquatic ecosystems. The present study provides information on microbial diversity in sediment contaminated by heavy metals from intensively farmed fish ponds. As well as, information on microbial taxa bioindicators of environmental quality of potential use in pollution monitoring and control. Most of the heavy metal concentrations did not exceed the UCC values, except for Cd and As which doubled the UCC concentrations. The CF values of the heavy metals studied reveal that Cu, Cr, Pb, Co, Ni, V and Sb contamination is low in the Pomacocha, Tipicocha and Tranca Grande lagoons. Cd contamination is moderate in the three lagoons and As contamination is considerable, except in Pomacocha where contamination is moderate. These results reveal pressure from fish farming activity, input of pollutants through tributaries running through areas with mining influence and runoff from agricultural areas^30–32^. In addition, they agree with other studies reporting that fish farming and environmental conditions around fish cages influence the sedimentation of these elements^33,34^. Heavy metal concentrations in the sediments of the studied lagoons are similar to those of other lagoons investigated in Peru^35,36^, but lower than those reported in aquatic ecosystems receiving high pollution loads^18,37^.

Analysis of microbial communities in lake sediments revealed that Proteobacteria are the most abundant and diverse phylum that play an important role in degradation and metabolism in lake sediments^38^. Other important phyla typical of freshwater ecosystems observed in this study are Cyanobacteria, Actinobacteria, Bacteroidetes, Firmicutes, Acidobacteria, Euryarchaeota, and Chloroflexi. This observation is in agreement with studies in similar environments in other regions that report the phyla Proteobacteria, Actinobacteria, Cyanobacteria, Bacteroidetes and Verrucomicrobia as the most abundant^39–41^. Proteobacteria (Alpha-, Beta- and Gammaproteobacteria) and Actinobacteria predominated in the sediment of the Pomacocha and Tranca Grande lagoons, which are in a mesotrophic state. Cyanobacteria is a phylum that performs photosynthesis, play a key role in nutrient cycling and are probably responsible for eutrophication processes in water bodies^42^. In this study, we found that Cyanobacteria predominate in sediments of the Tipicocha lagoon, which is in a mesotrophic-eutrophic state, a trophic state reported in previous studies^2^ This observation agrees with the study of Shen et al.^43^, who found large differences in the taxonomic structures of microbial communities in eutrophic, mesotrophic and oligotrophic aquatic environments. The Bacteroidetes and Verrucomicrobia found in the sediment of the studied lagoons have been widely reported in studies of aquatic environments contaminated by heavy metals^44^.

The results obtained for microbial diversity indices in lake sediments were lower than those reported in other studies using the same method of analysis^45^. This difference would be determined by multiple stressors such as heavy metal contamination of sediments in the studied ponds under fish pressure. It has been reported that there is a negative correlation between richness and heavy metals in inland lake sediments^46^. Other studies using the fingerprinting method reveal that the response of microbial communities to heavy metal contamination may vary according to the magnitude-dependent toxic effect^47^. Chemical contaminants can favor the proliferation of microbial consortia of more tolerant species that replace non-tolerant ones, increasing diversity. The use of different methods to determine richness may lead to different results, as they are limited to determine only the richness of dominant species.

The main contributions in community differentiation were made by the classes Alphaproteobacteria, Cyanophyceae, Betaproteobacteria, Actinobacteria and Deltaproteobacteria. These findings are similar to other studies that report these classes as the predominant classes in sediments of freshwater environments^48^. However, it differs with some studies regarding Alphaproteobacteria which report that they are generally not very abundant in freshwater environments^45,49^. Betaproteobacteria are diverse and commonly inhabit continental environments worldwide, where they are numerically dominant^50^. However, their abundance is determined by the depth of the water body, pH and nutrients^51^. Several studies have reported that nutrient sources are potential drivers of microbial community composition. Increased nutrient loading may favor select groups of bacteria that have the ability to rapidly consume these available resources or selectively eliminate certain bacteria from the community^52,53^. The reduction or absence of essential microorganisms in the nitrogen cycle will affect the nitrogen cycle chain and the transformation and removal of nitrogenous elements during bioremediation of eutrophic waters^54^. Other studies have shown that heavy metal contamination affects the abundance, composition and structure of microbial communities and disrupts replication processes by destroying microbial DNA^55,56^. The abundance of heavy metal susceptible microbes decreases as those resistant to these metal contaminants adapt and proliferate under chemical stress^49^.

Redundancy analysis revealed that As and V were the most important factors shaping microbial communities in lake sediments with intensive fish farming activity. The classes Alphaproteobacteria, Chitinophagia and Methanomicrobia observed in this study showed the greatest contribution to the structure of microbial communities in As-contaminated lagoons. This metalloid acts as a gene biotransforming agent that results in emergence and proliferation of resistance to this metalloid^40^, where organisms that are already tolerant become more competitive and thus more numerous. These three microbial classes can be used as bioindicators of As contamination. Oligoflexia, Ardenticatenia, Caldilineae, Methanomicrobia, Chloroflexia, Aquificae, Fimbriimonadia, Gemmatimonadetes, Sphingobacteriia, Acidimicrobiia and Nitrospira can be used to identify aquatic environments contaminated by Sb, Cr and Co, and Saprospiria from aquatic environments contaminated by V.

## Conclusions

This study is the first investigation of microbial diversity in lake sediment using metagenomic analysis in the Central Andes of Peru. Analysis of 16S rRNA gene amplicon sequences revealed that heavy metal contamination in sediment from intensively used fish lagoons modulates the composition and structure of microbial communities. SIMPER analysis showed Alphaproteobacteria, Betaproteobacteria and Actinobacteria as the classes with the highest percentage contributions to the differentiation of microbial communities. Diversity indices indicated that lagoons with lower anthropogenic pressure have higher microbial diversity, as is the case of Pomacocha lagoon. The dendrogram based on the similarity of Euclidean distances showed microbial communities from the most dominant to the rarest. The results presented here are the first direct comparison of microbial profiles of lake sediment with intensive fish farming and heavy metal effects in central Peru. The heavy metal contamination factor revealed that bacteria are prevalent in sediments with high levels of Cd and As. The RDA analysis determined that As and V were the influential factors in the composition of microbial communities. Finally, the results obtained allow us to expand the current knowledge of the composition of microbial communities in sediments of similar environments under fish pressure contaminated with heavy metals and to identify bioindicator taxa of environmental quality that can be used in the monitoring and control of heavy metal contamination.

## Methods

### Study area

**Figure 5:**
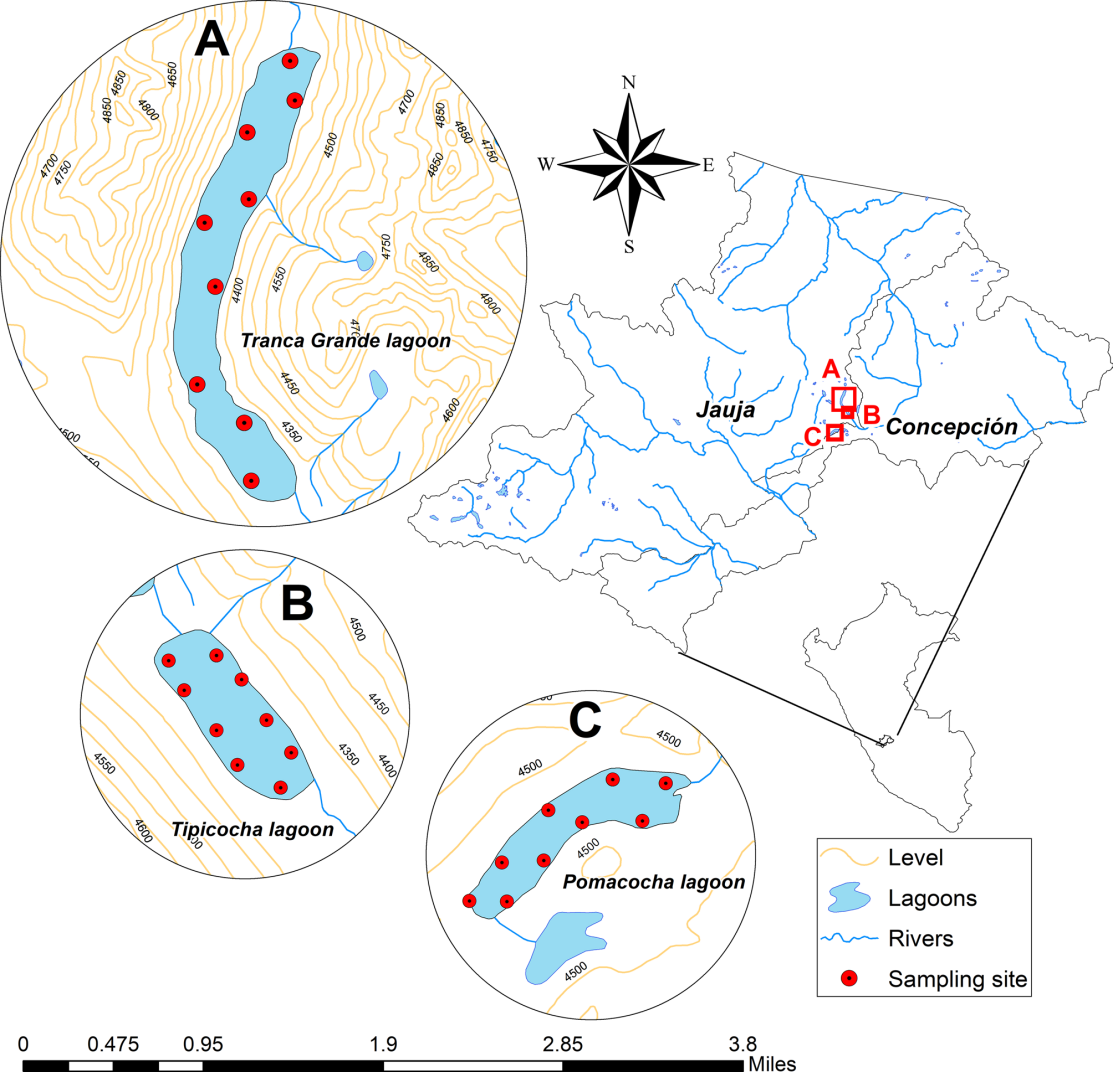
Location map of the study area in the Mantaro river watershed, Peru.

The lagoons considered in the study are located on the eastern slope of the upper Perene river basin in the Central Andes of Peru, between latitudes: 11° 4̍3′ 45S–12° 08′ 19S and longitudes: 75° 13′ 40W–75° 38′ 01W, at an altitude ranging from 4310 to 4330 masl. The study area was defined using ArcMap version 10.8 software^57^ (Fig. 5). The climate presents two contrasting periods, a dry period from May to September and a rainy period from October to April. Annual precipitation varies between 80 and 110 mm and the temperature ranges between − 2 and 16 °C^58^. The area and depth of the lagoons vary from 90 to 164 ha and from 9 to 25 m, respectively. Water temperature varies between 9.3 and 16.6 °C and turbidity between 7.3 and 10.18 NTU. Two lagoons are dominated by sandy sediment and the other by clayey sediment. These lagoons have been used for intensive culture of *Oncorhynchus mykiss* (rainbow trout) in large floating cages since the 1990s. The cages are constructed of eucalyptus wood or Guayaquil cane, nylon mesh and aluminum cylinders for volumes ranging from 56 m^3^ (for fry and juveniles) to 87.5 m^3^ (grow-out). Two of the three lagoons are in a mesotrophic state and one in a mesotrophic-eutrophic state^2^.

### Lake sediment sampling

Surface sediment samples (0–10 cm depth) were collected from three lagoons with intensive culture of *Oncorhynchus mykiss* in November 2019. In each lagoon, nine sampling stations were established covering the northern, central and southern part. At each sampling station, four sites were selected for sediment sampling. Sediment samples from each lagoon were collected using a stainless steel auger-type device. Samples from each station were mixed to obtain two 250 g composite samples. Sediment samples were packed in sterile airtight plastic bags and transported on ice to the laboratories for analysis. Sediment samples destined for microbial metagenomic analysis were stored at − 80 °C and for heavy metal analysis at 4 °C.

### Heavy metal determination, quality control and assurance

Heavy metals were extracted according to the standard method of environmental quality validated by INACAL of Peru (acronym of the National Institute of Quality in Spanish), using a mixture of HF, HNO_3_ and concentrated HClO_4_ (5:2:1). The reading was performed with an inductively coupled plasma mass spectrometer (ICP-MS, PerkinElmer NexION 1000). Quality control was performed by applying standard laboratory measurements and quality control methods including replication, the use of standards for each metal investigated and determination of instrument precision^59^. The determination of heavy metals was performed in triplicate, the blank experiments followed the same procedure applied for the samples.

Evaluation of the heavy metal contamination status. The contamination factor (CF) evaluates the heavy metal contamination status of the sediment^60^. The CF for each metallic element were calculated using the Eq. (1)^61^:

1 $$\mathrm{CF}=\frac{{\mathrm{C}}_{m} \, \mathrm{ sample }}{{\mathrm{C}}_{m} \, \mathrm{ background}}$$

where ‘C_m_ sample’ is the concentration of heavy metals in the sediment sample and ‘C_m_ background’ is the mean concentration of heavy metal present in the upper continental plate^28^.

### DNA extraction, PCR amplification of 16S rRNA genes, and sequencing

DNA extraction was performed from 0.5 g sample using the PrestoTM Soil DNA Extraction Kit, in accordance with the manufacturer's instructions and standard protocols. DNA concentration and quality were determined using a NanodropTM ONe quantification spectrophotometer (Thermo Fisher Scientific, Massachusetts, USA) obtaining ranges from 0.3 to 88.5 ng/µl^62^. PCR amplification was performed using the Gene One and GE Healthcare Life Sciences kits by mixing 1 µl of the 16S rRNA F universal primer, 1 µl of the 16S rRNA R universal primer, 22 µl of the PCR mix (containing premix buffer, MgCl2, dNTPs and taqPolymerase) and 1 µl DNA sample obtaining a total reaction volume of 25 µl. Primers 27 F (5′-AGAGTTGATCCTGGCTCAG-3′) and 1392R (5′-GGTACCTTGTACGACTT-3′) were used and amplified for a product of about 1365 bp. Bacterial sequencing of the 16S rRNA amplicon was performed using the standard next-generation Illumina MiSeq. The construction of the library was carried out commercially (ADMERA HEALTH LLC, USA).

### Bioinformatic analysis of sequence readings

The FASTQ files generated by the program FASTQC v0.11.9 were processed to know the length of the readings, the quality of the bases and the percentage of nucleotide bases, as we showed in a preliminary study in lake sediment bacterial communities^63^. Subsequently, quality filtering and removal of regions of the primer and adapters present in the readings was performed using the Trimmomatic v0.39 program^64^ with minimum trimming values of Q30 and trimming of readings below 30 bp. All individual reads were greater than 150,000 per isolate with a read length of 251 nucleotides and a quality value of each sequenced base greater than 30. The taxonomic analysis was performed using the program^65^, based on the database minikraken_20171019_4GB. This program also handles multiple scripts for circular representation. Finally, operational taxonomic units were identified and abundances calculated^66^.

### Statistical analysis

The KW test was used as a non-parametric method to compare heavy metal concentrations in sediment between the evaluated ponds. Tests were performed using R software. A probability of 0.05 or less is considered significant in testing the null hypothesis that there are no differences in concentrations and other calculated values^67^. The contribution of each species to the average Bray–Curtis dissimilarity among all groups was calculated using the SIMPER^68^. The heat map was generated using R4.0.5^69^ with the package pheatmap and clusters were performed according to the Bray–Curtis similarity analysis (data were square root transformed and fully linked to reduce the significance of extreme values) using the PAST program. Redundancy analysis (RDA) was used to investigate the relationships between sediment element concentrations and the distribution of OTU evaluated according to class in Canoco^70^. Linear discriminant analysis (LDA) effect size analysis (LEfSe) was performed using the Galaxy package to identify whether any individual taxa are discriminatory for the three gaps^71^.

### Nucleotide sequence access numbers

The 16S rRNA gene sequences reported in this study were sent to the GenBank database with the access number PRJNA657251 (https://www.ncbi.nlm.nih.gov/sra/PRJNA657251).

## Supplementary Information

Supplementary Information.
