# Author Correction: Microbial diversity in intensively farmed lake sediment contaminated by heavy metals and identification of microbial taxa bioindicators of environmental quality

**DOI:** 10.1038/s41598-022-07332-y

**Published:** 2022-02-23

**Authors:** María Custodio, Ciro Espinoza, Richard Peñaloza, Tessy Peralta‑Ortiz, Héctor Sánchez‑Suárez, Alberto Ordinola‑Zapata, Enedia Vieyra‑Peña

**Affiliations:** 1 Centro de Investigación de Medicina en Altura y Medio Ambiente, Facultad de Medicina Humana, Universidad Nacional del Centro del Perú, Av. Mariscal Castilla N° 3909, Huancayo, Peru; 2 Facultad de Ingeniería Pesquera y Ciencias del Mar, Universidad Nacional de Tumbes, Calle Los Ceibos S/N, Puerto Pizarro, Tumbes, Peru; 3 Departamento Académico de Medicina Veterinaria y Zootecnia, Facultad de Ciencias Agrarias, Universidad Nacional de Tumbes, La Cruz S/N, Tumbes, Peru

Correction to: *Scientific Reports* https://doi.org/10.1038/s41598-021-03949-7, published online 07 January 2022

The original version of this Article contained an error in Reference 32, which was incorrectly given as:

Haghnazar, H. et al. Chemosphere Potentially toxic elements contamination in surface sediment and indigenous aquatic macrophytes of the Bahmanshir River, Iran: Appraisal of phytoremediation capability. **285**, (2021).

The correct reference is listed below:

Haghnazar, H. *et al.* Potentially toxic elements contamination in surface sediment and indigenous aquatic macrophytes of the Bahmanshir River, Iran: Appraisal of phytoremediation capability. *Chemosphere* **285**, 131446 (2021).

The original Article has been corrected.

