# Supplementary material for: Microbial diversity in intensively farmed lake sediment contaminated by heavy metals and identification of microbial taxa bioindicators of environmental quality
Source: Sci Rep. 2022 Jan 7;12:80. doi: 10.1038/s41598-021-03949-7 (PMC8742047; doi:10.1038/s41598-021-03949-7)
Supplement: Supplementary file 1 — Supplementary Information. [file 41598_2021_3949_MOESM1_ESM.docx]

**Supplementary Information**

**Table S1**. Heavy metal contamination factor of lake sediment with intensive fish farming activity.

| **Sampling lagoon** | **Sample** | **Contamination Factor (CF)** | | | | | | | | | |
| --- | --- | --- | --- | --- | --- | --- | --- | --- | --- | --- | --- |
|  |  | As | Cd | Cu | Cr | Pb | Zn | Co | Ni | V | Sb |
| Pomacocha | 1 | 1.96 | 1.70 | 0.18 | 0.04 | 0.37 | 0.43 | 0.17 | 0.25 | 0.30 | 0.70 |
|  | 2 | 2.05 | 1.72 | 0.21 | 0.04 | 0.31 | 0.41 | 0.15 | 0.27 | 0.32 | 0.75 |
|  | 3 | 2.52 | 1.75 | 0.26 | 0.04 | 0.35 | 0.44 | 0.18 | 0.25 | 0.32 | 0.75 |
| Tipicocha | 1 | 5.04 | 1.85 | 0.33 | 0.05 | 0.35 | 0.52 | 0.22 | 0.52 | 0.46 | 0.85 |
|  | 2 | 4.57 | 1.77 | 0.29 | 0.05 | 0.35 | 0.49 | 0.22 | 0.47 | 0.45 | 0.90 |
|  | 3 | 4.29 | 1.91 | 0.39 | 0.05 | 0.33 | 0.51 | 0.20 | 0.55 | 0.50 | 0.80 |
| Tranca Grande | 1 | 3.27 | 1.74 | 0.44 | 0.04 | 0.32 | 0.52 | 0.17 | 0.53 | 0.54 | 0.80 |
|  | 2 | 2.99 | 1.71 | 0.47 | 0.04 | 0.33 | 0.51 | 0.17 | 0.58 | 0.49 | 0.75 |
|  | 3 | 2.99 | 1.92 | 0.39 | 0.04 | 0.36 | 0.50 | 0.17 | 0.56 | 0.52 | 0.85 |

**Table S2.** Relative abundance of microbial phyla in sediment contaminated by heavy metals from lagoons with intensive fish farming activity.

| **Phylum** | **Pomacocha** | **Tipicocha** | **Tranca Grande** |
| --- | --- | --- | --- |
| Proteobacteria | 46.50 | 54.44 | 53.21 |
| Cyanobacteria | 17.58 | 8.40 | 18.64 |
| Actinobacteria | 11.30 | 7.10 | 2.79 |
| Bacteroidetes | 8.68 | 9.00 | 10.31 |
| Firmicutes | 5.04 | 6.24 | 3.96 |
| Acidobacteria | 2.45 | 2.69 | 0.84 |
| Euryarchaeota | 2.14 | 2.33 | 2.19 |
| Chloroflexi | 1.88 | 1.85 | 2.43 |
| Ignavibacteriae | 1.21 | 1.16 | 1.54 |
| Dictyoglomi | 0.64 | 0.60 | 0.90 |
| Verrucomicrobia | 0.43 | 0.79 | 0.67 |
| Spirochaetes | 0.38 | 0.38 | 0.50 |
| Gemmatimonadetes | 0.34 | 1.49 | 0.54 |
| Deinococcus Thermus | 0.28 | 0.54 | 0.20 |
| Thermodesulfobacteria | 0.28 | 0.29 | 0.38 |
| Candidatus Saccharibacteria | 0.26 | 0.47 | 0.23 |
| Nitrospirae | 0.10 | 1.44 | 0.07 |
| Tenericutes | 0.10 | 0.16 | 0.11 |
| Chlamydiae | 0.09 | 0.07 | 0.05 |
| Caldiserica | 0.06 | 0.07 | 0.06 |
| Chlorobi | 0.06 | 0.12 | 0.04 |
| Planctomycetes | 0.05 | 0.05 | 0.12 |
| Fusobacteria | 0.04 | 0.05 | 0.05 |
| Synergistetes | 0.03 | 0.04 | 0.06 |
| Fibrobacteres | 0.03 | 0.02 | 0.03 |
| Candidatus Cloacimonetes | 0.02 | 0.06 | 0.04 |
| Armatimonadetes | 0.01 | 0.05 | 0.01 |
| Thermotogae | 0.01 | 0.01 | 0.02 |
| Deferribacteres | 0.003 | 0.003 | 0.00 |
| Kiritimatiellaeota | 0.003 | 0.002 | 0.002 |
| Thaumarchaeota | 0.002 | 0.054 | 0.002 |
| Aquificae | 0.001 | 0.016 | 0.006 |

**Table S3.** Similarity percentage analysis (SIMPER) at the microbial class level in sediment from lagoons with intensive fish farming activity contaminated by heavy metals

| Class | Av. dissim | Contrib. % | Cumulative % | Mean Pomacocha | Mean Tipicocha | Mean Tranca Grande |
| --- | --- | --- | --- | --- | --- | --- |
| Alphaproteobacteria | 4.30 | 19.08 | 19.08 | 6320 | 20400 | 11400 |
| Cyanophyceae | 3.37 | 14.96 | 34.04 | 20300 | 10500 | 21400 |
| Betaproteobacteria | 2.03 | 9.01 | 43.05 | 10800 | 10800 | 16900 |
| Actinobacteria | 1.70 | 7.55 | 50.6 | 7400 | 5040 | 1850 |
| Deltaproteobacteria | 1.36 | 6.04 | 56.65 | 19600 | 15700 | 15600 |
| Coriobacteriia | 1.13 | 5.02 | 61.67 | 4170 | 846 | 496 |
| Flavobacteriia | 1.05 | 4.66 | 66.33 | 6340 | 4230 | 7560 |
| Clostridia | 0.91 | 4.05 | 70.38 | 2780 | 5140 | 2160 |
| Chitinophagia | 0.91 | 4.04 | 74.43 | 322 | 3300 | 1670 |
| Nitrospira | 0.84 | 3.71 | 78.14 | 120 | 2840 | 80.7 |
| Acidobacteriia | 0.64 | 2.84 | 80.98 | 2980 | 3120 | 1040 |
| Methanomicrobia | 0.51 | 2.28 | 83.25 | 1620 | 3310 | 2140 |
| Gemmatimonadetes | 0.42 | 1.88 | 85.13 | 415 | 1810 | 619 |
| Gammaproteobacteria | 0.40 | 1.78 | 86.91 | 10300 | 10600 | 10800 |
| Dehalococcoidia | 0.28 | 1.25 | 88.16 | 994 | 683 | 1610 |
| Erysipelotrichia | 0.25 | 1.13 | 89.29 | 421 | 1240 | 464 |
| Bacteroidia | 0.23 | 1.02 | 90.31 | 1490 | 741 | 1100 |
| Saprospiria | 0.22 | 0.96 | 91.26 | 18.7 | 522 | 718 |
| Acidimicrobiia | 0.16 | 0.71 | 91.97 | 87 | 593 | 66.7 |
| Bacilli | 0.16 | 0.70 | 92.67 | 1550 | 1440 | 1090 |
| Dictyoglomia | 0.14 | 0.63 | 93.31 | 705 | 639 | 1100 |
| Deinococci | 0.14 | 0.60 | 93.91 | 290 | 708 | 276 |
| Thermoleophilia | 0.13 | 0.59 | 94.49 | 198 | 472 | 40 |
| Methanobacteria | 0.13 | 0.56 | 95.05 | 450 | 40.7 | 133 |
| Anaerolineae | 0.11 | 0.50 | 95.55 | 738 | 1040 | 980 |
| Epsilonproteobacteria | 0.11 | 0.47 | 96.02 | 849 | 1180 | 1000 |
| Ignavibacteria | 0.10 | 0.45 | 96.47 | 1450 | 1630 | 1650 |
| Cytophagia | 0.09 | 0.40 | 96.87 | 136 | 228 | 427 |
| Verrucomicrobiae | 0.08 | 0.34 | 97.21 | 232 | 476 | 364 |
| Methylacidiphilae | 0.06 | 0.28 | 97.48 | 223 | 385 | 180 |
| Thermodesulfobacteria | 0.05 | 0.24 | 97.72 | 327 | 441 | 468 |
| Ardenticatenia | 0.05 | 0.21 | 97.93 | 140 | 298 | 217 |
| Oligoflexia | 0.05 | 0.20 | 98.14 | 3.33 | 153 | 89.7 |
| Spirochaetia | 0.05 | 0.20 | 98.34 | 467 | 469 | 580 |
| Planctomycetia | 0.04 | 0.17 | 98.51 | 67.3 | 52 | 179 |
| Rubrobacteria | 0.04 | 0.16 | 98.67 | 57.7 | 141 | 20.3 |
| Thermoplasmata | 0.03 | 0.12 | 98.8 | 450 | 393 | 387 |
| Solibacteres | 0.03 | 0.12 | 98.92 | 88 | 71.3 | 2 |
| Mollicutes | 0.03 | 0.12 | 99.03 | 110 | 196 | 147 |
| Sphingobacteriia | 0.03 | 0.11 | 99.14 | 24.3 | 96 | 12.7 |
| Spartobacteria | 0.02 | 0.10 | 99.25 | 7.67 | 22.3 | 84 |
| Chlorobia | 0.02 | 0.08 | 99.33 | 69.3 | 120 | 64.3 |
| Negativicutes | 0.02 | 0.08 | 99.4 | 341 | 296 | 330 |
| Chlamydiia | 0.02 | 0.07 | 99.47 | 115 | 78 | 63.3 |
| Synergistia | 0.02 | 0.07 | 99.54 | 44.3 | 47.3 | 92.3 |
| Fimbriimonadia | 0.01 | 0.06 | 99.6 | 0.0 | 46.3 | 7.0 |
| Gloeobacteria | 0.01 | 0.06 | 99.66 | 7.7 | 15.0 | 48.7 |
| Acidithiobacillia | 0.01 | 0.05 | 99.71 | 29.0 | 36.7 | 64.7 |
| Opitutae | 0.01 | 0.04 | 99.75 | 7.7 | 35.3 | 14.7 |
| Chloroflexia | 0.01 | 0.03 | 99.78 | 4.0 | 29.0 | 9.3 |
| Caldisericia | 0.01 | 0.03 | 99.81 | 83.0 | 70.0 | 74.0 |
| Fibrobacteria | 0.01 | 0.03 | 99.84 | 32.3 | 21.0 | 43.0 |
| Thermomicrobia | 0.01 | 0.03 | 99.87 | 11.0 | 30.7 | 30.0 |
| Caldilineae | 0.01 | 0.02 | 99.9 | 0.3 | 18.3 | 10.3 |
| Fusobacteriia | 0.01 | 0.02 | 99.92 | 50.7 | 66.3 | 61.3 |
| Aquificae | 0.01 | 0.02 | 99.94 | 2.0 | 19.0 | 6.0 |
| Chthonomonadetes | 0.00 | 0.02 | 99.96 | 21.3 | 22.7 | 14.0 |
| Thermotogae | 0.00 | 0.01 | 99.97 | 14.3 | 10.7 | 18.7 |
| Deferribacteres | 0.00 | 0.01 | 99.98 | 6.7 | 4.3 | 0.0 |
| Tissierellia | 0.00 | 0.01 | 99.99 | 3.0 | 6.0 | 0.3 |
| Kiritimatiellae | 0.00 | 0.00 | 99.99 | 3.0 | 1.0 | 0.7 |
| Limnochordia | 0.00 | 0.00 | 100 | 1.3 | 0.0 | 1.7 |
| Thermoprotei | 0.00 | 0.00 | 100 | 0.0 | 0.0 | 1.3 |
| Nitrososphaeria | 0.00 | 0.00 | 100 | 1.3 | 0.0 | 0.3 |
| Phycisphaerae | 0.00 | 0.00 | 100 | 0.0 | 0.3 | 0.0 |
| Blastocatellia | 0.0000 | 0.0000 | 100 | 0 | 0 | 0 |

**Table S4.** Analysis 'Distance-based-RDA-forwsel', step 'db-RDA Forward selection' (Total variation is 0.22805, explanatory variables account for 99.0% (adjusted explained variation is 91.6%).

| Summary Table: | | | | |
| --- | --- | --- | --- | --- |
| Statistic | Axis 1 | Axis 2 | Axis 3 | Axis 4 |
| Eigenvalues | 0.54 | 0.42 | 0.02 | 0.01 |
| Explained variation (cumulative) | 53.98 | 95.61 | 98.01 | 98.65 |
| Pseudo-canonical correlation | 1.00 | 1.00 | 1.00 | 0.88 |
| Explained fitted variation (cumulative) | 54.55 | 96.62 | 99.05 | 99.70 |
| Analysis 'Distance-based-RDA-forwsel', step 'db-RDA Forward selection' | | | | |
| Forward Selection Results: | | | | |
| Name | Explains % | Contribution % | pseudo-F | P |
| Arsenic | 50.1 | 50.1 | 7 | 0.002 |
| Vanadium | 39 | 39 | 21.4 | 0.008 |
| Chromium | 2.7 | 2.7 | 1.7 | 0.178 |
| Nickel | 1.9 | 1.9 | 1.2 | 0.394 |
| Antimony | 2.2 | 2.2 | 1.6 | 0.214 |
| Cobalt | 1.3 | 1.3 | 1 | 0.448 |
| Zinc | 1.7 | 1.7 | 1.6 | 0.436 |
| Correlation between explanatory variables and the case score derived from response variables position | | | | |
|  | CorE.1 | CorE.2 | CorE.3 | CorE.4 |
| Arsenic | -0.9157 | -0.3369 | -0.1037 | 0.0303 |
| Cadmium | -0.5695 | -0.0408 | -0.0169 | -0.0306 |
| Copper | -0.6491 | 0.6441 | 0.2146 | 0.2521 |
| Chromium | -0.804 | -0.4845 | 0.1241 | 0.0471 |
| Lead | 0.1277 | -0.301 | -0.5982 | 0.1763 |
| Zinc | -0.884 | 0.308 | -0.0525 | 0.0772 |
| Cobalt | -0.6801 | -0.6119 | -0.3153 | 0.1604 |
| Nickel | -0.8787 | 0.4352 | 0.108 | 0.0725 |
| Vanadium | -0.8298 | 0.511 | 0.0152 | 0.0069 |
| Antimony | -0.8065 | -0.0791 | -0.4718 | -0.0496 |


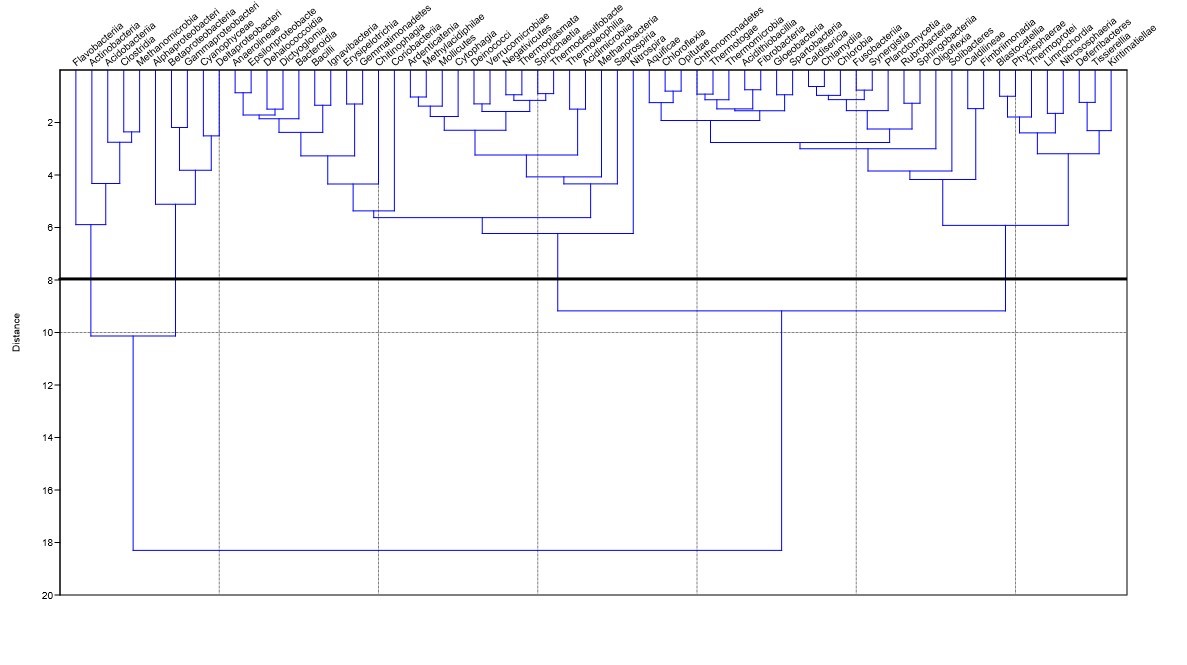


**Figure S1.** Linkage-based dendrogram showing hierarchical clustering patterns of 65 microbial classes into four groups with measured Euclidean distances.
